# Point-of-care C-reactive protein test results in acute infections in children in primary care: an observational study

**DOI:** 10.1186/s12887-022-03677-5

**Published:** 2022-11-04

**Authors:** Liselore De Rop, Tine De Burghgraeve, An De Sutter, Frank Buntinx, Jan Y Verbakel

**Affiliations:** 1 EPI-Centre, Department of Public Health and Primary Care, KU Leuven, Leuven, Belgium; 2 Department of Family Practice and Primary Health Care, Ghent University, Ghent, Belgium; 3 Research Institute Caphri, Maastricht University, Maastricht, The Netherlands; 4 NIHR Community Medtech and IVD cooperative, Nuffield Department of Primary Care Health Sciences, University of Oxford, Oxford, UK

**Keywords:** Child, Point-of-care systems, C-reactive protein, Acute illness, Serious infections, Primary care

## Abstract

**Background:**

Acute infections are a common reason for children to consult primary care. Serious infections are rare but differentiating them from self-limiting illnesses remains challenging. This can lead to inappropriate antibiotic prescribing. Point-of-care C-reactive protein testing is used to guide antibiotic prescribing in adults. However, in children its use remains unclear. The purpose of this study was to assess point-of-care CRP test levels with respect to patients’ characteristics, care setting, preliminary diagnosis, and management.

**Methods:**

A prospective observational study was performed in children with an acute infection presenting to ambulatory care in Belgium.

**Results:**

In this study 8280 cases were analysed, of which 6552 had a point-of-care CRP value available. A total of 276 physicians participated. The median patient age was 1.98 years (IQR 0.97 to 4.17), 37% of children presented to a general practitioner, 33% to a paediatric out-patient clinic, and 30% to the emergency department. A total of 131 different preliminary diagnoses were found, with acute upper airway infection as the most frequent. In 6% (n = 513) patients were diagnosed with a serious infection. The most common serious infection was pneumonia. Antibiotics were prescribed in 28% (n = 2030) of all episodes. The median CRP over all infectious episodes was 10 mg/L (IQR < 5–29). Children below 5 years of age and those presenting to a paediatrician had a higher median CRP. Median CRP in patients with serious infections was 21 mg/L (IQR 6 to 63.5). Pneumonia had a median CRP of 48 mg/L (IQR 13–113). In the episodes with antibiotics prescription, median CRP level was 29 mg/L (IQR 10–58) compared to 7 mg/L (IQR < 5–19) when they were not prescribed.

**Conclusion:**

A low POC CRP as a standalone tool did not seem to be sufficient to rule out serious infections, but its potential in assessing serious infections could increase when integrated in a clinical decision rule.

**Trial registration:**

ClinicalTrials.gov Identifier: NCT02024282 (registered on 31/12/2013).

**Supplementary Information:**

The online version contains supplementary material available at 10.1186/s12887-022-03677-5.

## Background

In primary care, infectious disease in children is very common. Most common are non-serious, self-limiting infections. Less than 1% will have a serious infection [1]. However, serious infections can be associated with both significant morbidity and mortality [1, 2]. Therefore, recognition remains crucial. Nevertheless, differentiating serious from non-serious infections can be difficult. In an early stage signs and symptoms can be very alike in both groups [3–5]. Studies show that at first contact many serious infections, with up to half of children with meningococcal disease, are not recognized [6, 7]. Furthermore, diagnostic uncertainty can lead to unnecessary additional testing, referral, or inappropriate antibiotic prescribing [8, 9].

Antimicrobial resistance is a growing threat and will not only result in unnecessary exposure to side effects and increased healthcare costs, but also increased morbidity and mortality [9, 10]. A recent study estimated 1.27 millions deaths attributable to antimicrobial resistance in 2019 [11].

During 2014, 38% of all Belgian children were given at least one prescription of antibiotics in outpatient care [12]. When children consulted their general practitioner (GP) with a respiratory tract infection, a Belgian GP-based continuous and integrated computerized morbidity registration network (INTEGO) showed that about 45% received an antibiotic prescription [13].

Expectations of the child’s parents and an inadequate communication can contribute to overprescribing. [14] Furthermore, physicians do not want to deny antibiotics to a child with a possible bacterial infection [15–19]. To help physicians in prescribing antibiotics appropriately, evidence-based guidelines were created 20. However, these guidelines are not always clear-cut, leaving room for doubt or subjective assessment [21].

A biomarker can help distinguish serious infections and reduce antibiotic prescriptions. Testing C-reactive protein (CRP) is used in ambulatory care to differentiate between serious and non-serious diseases. CRP is an acute-phase protein that increases in response to inflammation or tissue damage [22]. However, delay between blood sampling and receipt of the results can be an important barrier [8]. Furthermore, determining it in acutely ill children can be an obstacle, as it requires a venous blood sample. Point-of-care (POC) testing of CRP, being minimally invasive by using a finger prick, can be considered a good alternative. Research in adults has previously shown that POC CRP testing can safely reduce antibiotics prescriptions in upper respiratory tract infections (URTI) [9, 23, 24]. However, the role of the POC CRP test in children remains unclear, as strict cut-off values are yet to be determined. Therefore, the aim of this study is twofold: (1) assessment of POC CRP level in relation to the diagnosis of acute infections in children in ambulatory care and (2) assessing whether it can be a good candidate to help differentiate between serious and non-serious infection in children.

## Methods

### Study design and setting

This study was a prospective observational study performed in children with an acute infection in ambulatory care, defined as general practice, paediatric outpatient clinic, and emergency department (ED). The aim was to observe POC CRP test results in relation to patients’ characteristics, health care setting, preliminary diagnosis, and treatment actions. The focus was on the level of POC CRP and the preliminary diagnosis. The data used in this study was collected within the ERNIE 2 trial, a diagnostic accuracy study of which the protocol and the main results have been reported previously [3, 4, 10, 14, 25]. To avoid contamination allocation was performed at practice with a ratio of 1:1:1:1. The study included four allocation groups, family physicians (1) using a POC CRP test, (2) applying a brief intervention with safety net advice, (3) using POC CRP test plus applying a brief intervention with safety net advice, and (4) usual care [14]. The trial was registered on 31/12/2013, ClinicalTrials.gov Identifier: NCT02024282.

### Study population

#### Participants

The study included acutely ill children presenting to a GP or paediatrician with a new acute illness episode of maximum five days in Flanders, Belgium. Children aged 1 month to 16 years were consecutively recruited. Only acute episodes during the first encounter were included, children who were referred to secondary care were excluded. When the physician included the same child twice within five days, this was considered a repeated measure and discarded from the analysis. If the acute illness was caused by purely traumatic or neurological conditions, intoxication, a psychiatric problem, or an exacerbation of a known chronic condition the child was excluded. Written informed consent was obtained from the child’s parent or legal guardian. Physicians could participate if they were able to recruit children consecutively during the inclusion period. Details of the recruitment procedure can be found in the published study protocols [3, 14].

### Intervention

The Afinion CRP test (the Afinion AS100 Analyzer, Alere, USA) was used to perform the POC CRP test [3, 26]. Children at risk for severe infection, based on testing positive on a 5-stage decision tree [25, 27], always got tested and children testing negative on this decision tree either got a POC CRP test or usual care, depending on their per practice cluster randomization [3, 14]. All physicians were trained on how to use the device. The Afinion CRP test can measure CRP values from 5 mg/L up to 200 mg/L. If CRP is below 5 mg/L it is displayed as < 5 mg/L, if it is above 200 mg/L as > 200 mg/L. The physicians were not given any guidance on how to interpret the CRP-results, as reliable cut-off values for children in primary care were unavailable at the time [28].

### Ethical approval

The ethical Review Board of the University Hospitals/KU Leuven approved the protocol of this trial under reference S-number S54664. The study was conducted according to the approved protocol and the principles outlined in the Declaration of Helsinki. Written informed consent was obtained from the child’s parent or legal guardian.

### Statistical analysis

Calculation of the sample size was described previously [3, 14]. Descriptive analyses (median, interquartile ranges, …) were performed using Excel (Microsoft Corporation, Redmond, USA) and SPSS (version 26; SPSS inc., Chicago, Illinois, USA). Only complete case analyses were performed, because the assumption of missing at random was not fulfilled to allow for multiple imputation of missing data.

The patients’ characteristics studied were age, sex, and the healthcare setting: GP, paediatric out-patient clinics, and ED.

Physicians were asked to register the preliminary diagnoses as free text. These diagnoses were afterwards classified by one author (LDR) and checked by a second author (JV) using the second edition of the International Classification of Primary Care (ICPC-2) [29]. Any disagreement between both authors was resolved through discussion. Whenever a physician added a question mark, a differential diagnosis, or the word ‘presumable’, we assumed there was uncertainty surrounding the chosen diagnosis.

If, after the first encounter, a patient was admitted to the hospital for more than 24 h within 5 days after initial presentation, this was considered a serious infection. In these children the data considering POC CRP of the first encounter with GP or paediatrician at out-patient clinic or ED was analysed. Following serious infections were included, as previously described within the ERNIE 2 trial: pneumonia, complicated urinary tract infection (UTI), sepsis, meningitis, appendicitis, cellulitis, bacterial gastroenteritis, osteomyelitis, viral respiratory tract infection complicated by hypoxia, gastroenteritis with dehydration (DH), and abscess [3, 4]. Pneumonia can sometimes be treated safely at home with antibiotics, for the serious infections in our study we therefore choose to consider only those episodes of pneumonia for which hospitalization was needed.

Physicians registered whether they prescribed antibiotics or not and whether this was an immediate or delayed prescription. They also recorded their beliefs about parent’s expectation towards antibiotics. Additional examinations and referral were registered.

## Results

### Participant flow, recruitment and numbers analysed

Acute infectious episodes were registered between January 2013 and February 2014. A total of 8280 episodes was analysed. Participant flow, including losses and exclusions after randomisation, was described previously [4, 10]. The trial was ended per protocol.

### Baseline characteristics

In 53,4% (n = 4396) of infectious episodes it concerned boys. The median age was 1.98 years (IQR 0.97 to 4.17). Baseline characteristics for each group were described previously [4, 25]. As per protocol the CRP value was not measured in 1728 cases. Age, sex, and the ranking of frequency of preliminary diagnosis were similar in the group with measured CRP value compared to all children recruited. The majority of children was not known with a chronic condition (n = 7827). In 5.5% (n = 453) of cases a chronic condition was registered, most frequently lung disease, including asthma. In 36.9% (n = 3057) of cases children presented to the GP, 32.9% (n = 2725) to paediatric out-patient clinic and 30.2% (n = 2498) to the ED. The physician’s characteristics are described elsewhere [10, 25].

### Preliminary diagnoses

Preliminary diagnoses are shown in **Appendix 1**. A total of different 131 ICPC-2 codes were found. In accordance with the ICPC-2 code system, acute URTI (n = 1536) was diagnosed most frequently, followed by viral disease other (n = 1284), acute bronchitis/bronchiolitis (n = 918), acute otitis media/myringitis (n = 741) and gastroenteritis presumed infection (n = 654). Pneumonia (n = 411) and influenza (n = 297) held the sixth and seventh place, respectively. In 82.2% (n = 6809) of all infectious episodes only one diagnosis, and in 17.7% (n = 1471) more than one diagnosis was registered. Physicians were unsure about the preliminary diagnosis in 8.8% (n = 730) of cases. In 6.2% (n = 513) of cases patients were diagnosed with a serious infection. The different serious infections are shown in Table 1. Most common serious infections were pneumonia, gastroenteritis with DH and complicated UTI.

**Table 1:** Serious infections and POC CRP values

Serious infection	n	CRP (mg/L)	Median	Min	Max	IQR
**All**	484		21	< 5	> 200	6–64
**Pneumonia**	164		48	< 5	> 200	13–113
**Gastro-enteritis with DH**	162		9.5	< 5	> 200	< 5–30
**Complicated UTI**	58		54.5	< 5	> 200	22–127
**Viral RTI with hypoxia**	53		9	< 5	48	< 5–14
**Meningitis**	15		10	< 5	76	< 5–39
**Other**	15		18	< 5	> 200	7–25
**Cellulitis**	6		27	< 5	150	< 5–71
**Bacteraemia**	4		52	17	138	20–123
**Abscess**	4		10	< 5	17	5–16
**Sepsis**	3		31	< 5	75	< 5-/

*Overview of the different serious infections with reported POC CRP values described by prevalence, median POC CRP and IQR. If a patient was admitted to the hospital for more than 24 h, it was considered a serious infection. POC, point-of-care; CRP, C-reactive protein; DH, dehydration; UTI, urinary tract infection; RTI, respiratory tract infection*

### Management

Antibiotics were prescribed in 2030 (27.7%) episodes, with a delayed prescription in 29.3% (n = 601) of them. Physicians thought parents expected antibiotics in 684 (11.2%) episodes, in 68.9%(n = 471) of these cases antibiotics were prescribed. In the GP setting antibiotics were prescribed in 30.4% (n = 856) of cases, in paediatric outpatient clinic in 27.5% (n = 649) and in the ED in 24.2% (n = 525). Patients with acute URTI were prescribed antibiotics in 18.7% (n = 355) of cases. In the GP setting, in the paediatric outpatient clinic, and in the ED antibiotics were prescribed in URTI in respectively 19.1% (n = 170), 20.1% (n = 108), and 16.3% (n = 77) of cases. Physicians were asked to justify their antibiotic prescribing. Different reasons were given, such as: a specific diagnosis (e.g. otitis, pneumonia, tonsillitis), high CRP-value, duration of fever or illness, seriously ill child, findings on clinical examination, medical history, additional testing (e.g. x-ray, pcr), expectation of the parents, young age of the child, and timing of consultation (e.g. before the weekend). The reasons over the three settings were similar. (**Appendix 2**) Additional examination was performed in 39.7% (n = 2925).

### POC CRP

The median POC CRP over all infectious episodes was 10 mg/L (IQR < 5–29).

#### Patients’ characteristics

**Graph 1:**
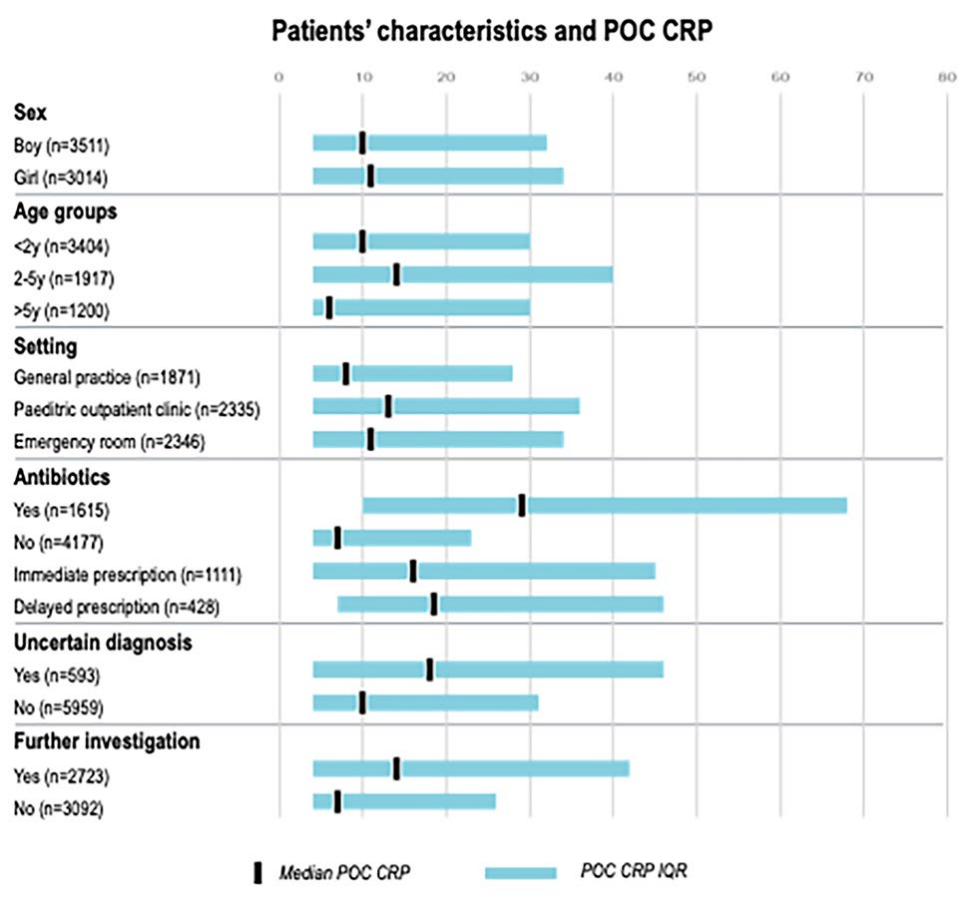
Median POC CRP (mg/L) and IQR for different patients’ characteristics, including sex, age groups, and healthcare setting, antibiotic prescribing, uncertain diagnosis, and further investigation. Y, years; POC, point-of-care; CRP, C-reactive protein; IQR, interquartile range

We looked at median POC CRP value in comparison to sex, age group, and the healthcare setting. This is displayed in Graph 1. Children below 5 years old had a higher median CRP compared to older children (11 mg/L, IQR < 5–29 vs. 6 mg/L, IQR < 5–26). In comparison to GP (8 mg/L, IQR < 5–24) a higher median CRP was seen in the paediatric outpatient clinic (13 mg/L, IQR < 5–32) and ED (11 mg/L, IQR < 5–30).

#### Preliminary diagnoses and serious infections

**Graph 2:**
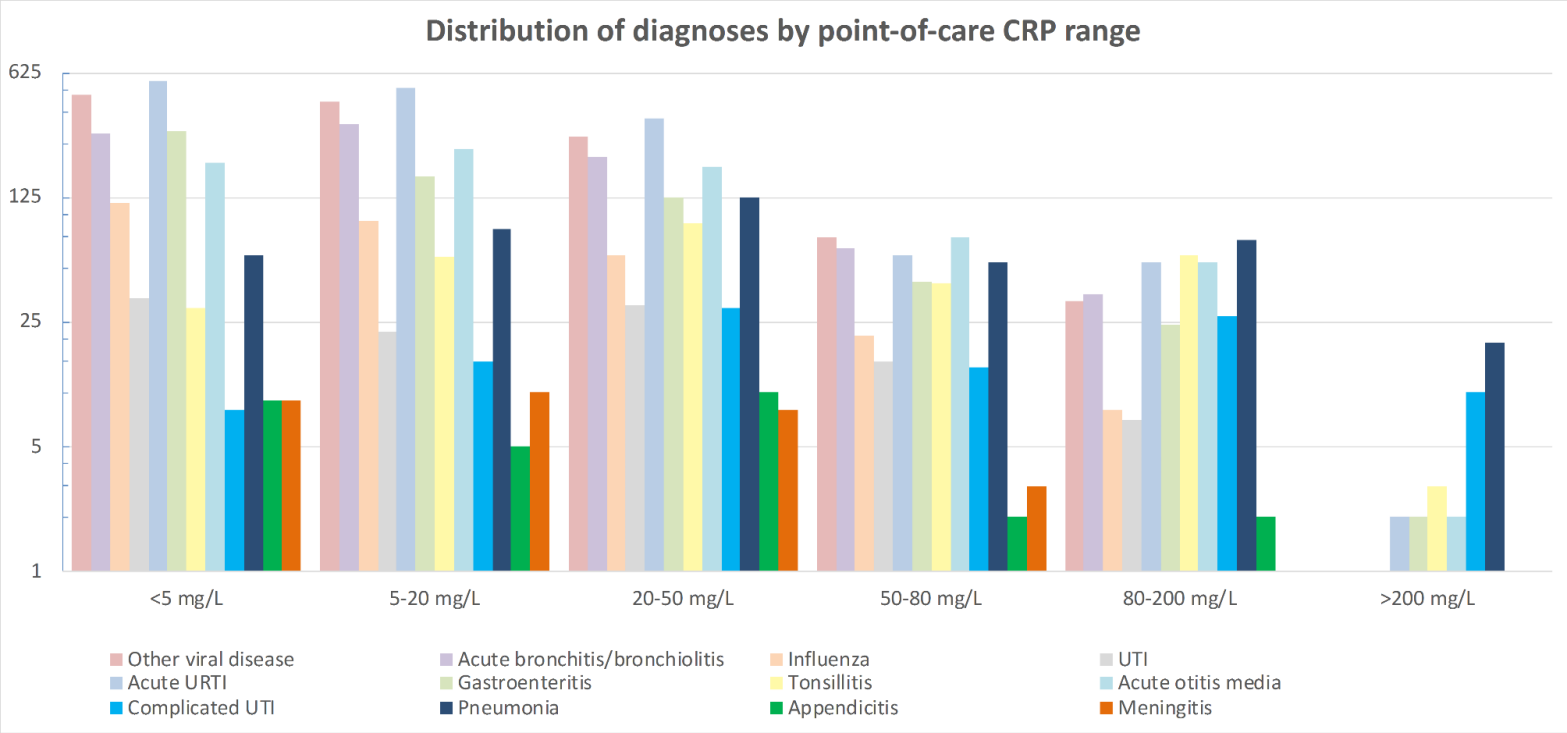
Distribution of frequency of diagnoses by point-of-care CRP range. X-axis displays 5 different point-of-care CRP ranges. Y-axis displays the frequency of the diagnoses on a logarithmic scale. CRP, C-reactive protein; URTI, upper respiratory tract infection; UTI, urinary tract infection

**Graph 3:**
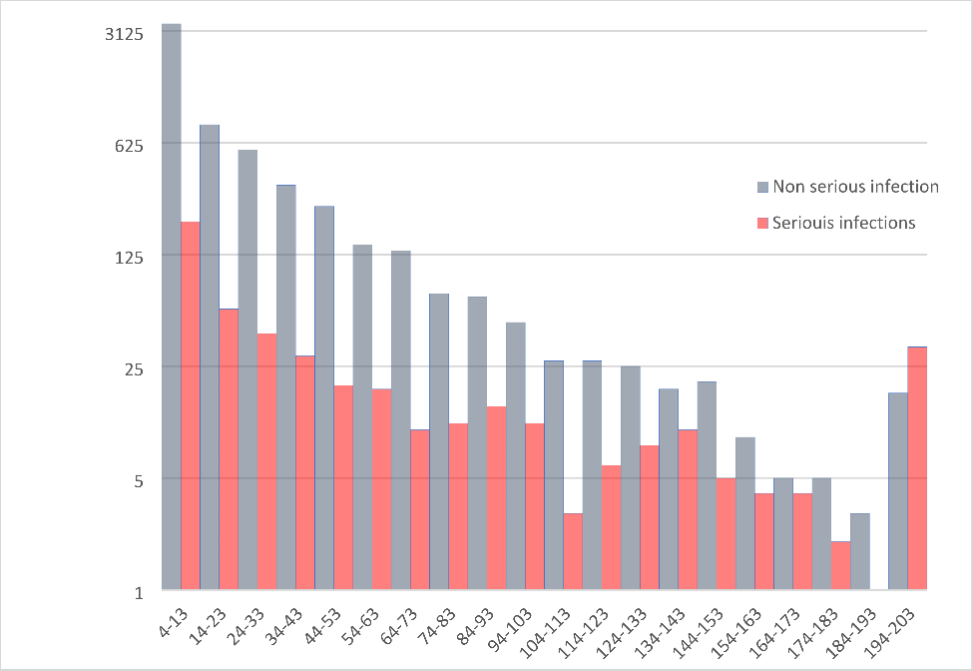
POC CRP distribution in serious and non-serious infection. This graph shows the distribution of the frequency of POC CRP value (mg/L) in non-serious and serious infections. X-axis displays the POC CRP range (mg/L), and the Y-axis displays the absolute frequency on a logarithmic scale. POC, point-of-care; CRP, C-reactive protein

The distribution of different ICPC-2 codes and their CRP-range is shown in Graph 2 and **Appendix 1**. We found 513 cases of serious infections, in 29 of them a CRP value was missing. If the patient had a serious infection the median CRP was 21 mg/L (IQR 6-63.5). Serious infections in the GP setting had a median CRP of 8.5 mg/L (IQR < 5-28.5), in the paediatric outpatient clinic 35 mg/L (IQR 9-107) and in the ED 21 mg/dL (IQR 5-54.5). In the group without serious infection, median CRP was 10 mg/L (IQR < 5–27). This is displayed in Graph 3. Most common serious infection was pneumonia (n = 164), with a median CRP of 48 mg/L (IQR 13–113), followed by gastroenteritis with DH (n = 162, median CRP 9.5 mg/L, IQR < 5–30) and complicated UTI (n = 58, median CRP 54.5 mg/L, IQR 22–127).

#### Management

A higher median POC CRP was seen when physicians requested additional testing and when antibiotics were prescribed. (Graph 1).

## Discussion

### Main findings and interpretation

Most children included in our study were below five years of age. In this group we observed a higher median POC CRP compared to older children. It is known that overall prevalence of infection as well as serious infection, is higher in young children [1]. Differentiating between serious and non-serious infection is important. We observed that serious infections tended to have higher CRP values, however serious infections with CRP < 5 mg/L were also found. The highest number of patients with pneumonia was found in the CRP range of 20 to 50 mg/L. A serious infection with a low CRP can be explained by the fact that in the early stage of disease the inflammatory response is still developing and CRP is still low [30]. The study of Putto et al. [31], examining serum CRP concentrations in 154 febrile children, found that when CRP value was less than 20 mg/L and duration of disease more than 12 h with no identifiable focus of bacterial infection, all children could be classified as having a viral infection. CRP values of 20–40 mg/L were recorded in both viral and bacterial infections and most febrile children with CRP ≥40 mg/L had a bacterial infection. Other studies looked at the relation between CRP and pneumonia. The meta-analysis of Flood et al. [32] found that serum CRP exceeding 40–60 mg/L weakly predicts a bacterial aetiology of pneumonia. The study of Koster et al. [33], performed in children presenting to the ED with suspected pneumonia, found CRP level to have an independent diagnostic value for pneumonia in this setting, however low levels did not exclude pneumonia. The study of Marcus et al. [34] found POC CRP to be a useful predictor of bacterial pneumonia in children in the ED, as significantly higher CRP levels were associated with bacterial than with viral pneumonia. On the other hand, a Finnish study in primary care found no significant association in CRP values with the aetiology of pneumonia [35]. In our data we did not differentiate between bacterial and viral pneumonia, but we similarly observed that higher CRP tends to be associated with pneumonia, but that lower CRP might not exclude it.

Median POC CRP was higher when children were seen by a paediatrician, both at the paediatric outpatient clinic and the ED. As previously mentioned, infections are more prevalent in young children [1]. Furthermore, access to specialist care is decent in Belgium, and parents with very young children tend to consult the paediatrician instead of their GP [1]. Moreover, it is highly likely that parents assuming their child to be serious ill, directly consult the paediatrician. This difference should be investigated further.

In our study the antibiotic prescribing rate was 27.7%. However, it is more than 10% lower compared to the antibiotic prescribing rate in all Belgian children in outpatient care in 2014 [12]. The antibiotic prescribing rate was higher in the GP setting (30.4%) compared to the paediatric outpatient clinic (27.5%) and the ED (24.2%). In 18.7% of cases with acute URTI, antibiotics were prescribed. This is lower than the findings of the Belgian GP-based registration network INTEGO (45%) [13]. In a previous part of our study it was shown that normal levels of point-of-care CRP reduced the level of antibiotic prescribing [21]. At the GP and paediatric outpatient setting prescribing rate in acute URTI were similar, respectively 19.1% and 20.1%, in the ED however it was 16.3%. In a recent study the pharmacy dispensing data on antibiotics in Belgium were analysed and it was found that GPs prescribed the most antibiotics [36]. Different reasons exist for inappropriate antibiotic prescribing, such as diagnostic uncertainty [8, 9]. This can be a reason for the higher prescribing rate in GPs, since in the ED a more extensive workup is available, reducing the diagnostic uncertainty. Furthermore, parents’ expectation might influence the GP, not wanting to harm the doctor-patient relation [14].

### Strengths and limitations

This trial was a multicentre study, simultaneously recruiting in 3 different settings, including 8280 observations. Due to the relative low prevalence of serious infections in primary care, this large cohort is an important strength. Furthermore, the study was performed in primary care, whereas previous studies focused more on high-prevalence settings. We did not apply any age or symptom criteria, and POC CRP testing was performed regardless of the clinical presentation. Therefore, a large heterogenic group was studied, representing a real-life situation, and the risk of selection bias was limited. This made it possible to assess the value of CRP in different diagnoses, non-severe and severe. However, in clinical practice use of POC CRP will be different, possibly based on clinical assessment. To represent a real-life situation, we did not perform repeated measurements of POC CRP on different time points. However, studies have shown that a single low CRP measurement can underestimate the diagnosis of a serious infection, and therefore suggest relying on one or more repeated measurements [37, 38].

Furthermore, as time since start of illness was not addressed in this paper, this could be a limitation considering POC CRP values can vary during an acute illness episode. However, when analyzed in relation with duration of fever, POC CRP did not differ significantly over time in our study. (**Appendix 3**).

Only acute episodes during the first encounter and preliminary diagnoses were included. Information on additional testing was collected, however, in non-serious cases, the final diagnosis was not defined. This approach reflects a real-life situation, it is however a limitation that we cannot verify the final diagnosis with regard to the POC CRP value. In cases of serious infections, however, the final diagnosis was available. To avoid diagnostic labelling, when a specific preliminary diagnosis is registered to support prescribing antibiotics, physicians were asked to justify their antibiotic prescription. The study depends on the quality of the registered information.

### Practical implementation and implications for research

Studies show that POC CRP testing is accurate, reliable, and user-friendly in children [26, 39]. The study of Jones et al. [40] found that GPs would like to use POC CRP testing if strong evidence of the impact on patient care is available. Increased levels of POC CRP can be associated with serious infections, however we do not recommend physicians to solely base their clinical decision on POC CRP. Low POC CRP levels do not necessarily rule out serious infections. However, when combined with a clinical decision tool its potential increases. A previous analysis of our study [4], combining POC CRP with a clinical decision tool, shows that in children with increased risk (presenting with breathlessness, temperature ≥40 °C, diarrhoea in children aged 12–30 months or clinician’s gut feeling), CRP levels < 5 mg/L can safely rule out serious infection. In adults multiple studies show that POC CRP testing has a positive effect on the antibiotic prescribing rate [23, 41–43]. POC CRP testing is therefore now recommended in NICE and Dutch guidelines for acute cough in adults [44, 45]. For children the situation remains somewhat unclear, as clear cut-off values of CRP are lacking and in comparable health care settings, no significant reduction of the prescribing rate is seen [10, 28, 46]. A study in Vietnam, consisting of 1000 children, did show a significant effect of POC CRP testing on the antibiotic prescribing rate [47] We notice that the prescribing rate in both groups, POC CRP testing and routine care, was high, respectively 68% and 77%. This might explain the effect of POC CRP testing, as studies show that in GPs who already have a low antibiotic prescribing rate, POC CRP testing does not reduce it [48]. A more recent study of Lemiengre et al. [10] showed that systematic POC CRP without guidance in comparison to usual care did not influence antibiotic prescribing in children with acute non-severe infections in primary care, and a Norwegian study [49], which took place in out-of-hours services, found that pre-consultation screening with CRP in children with fever and/or respiratory symptoms did not significantly affect the prescription of antibiotics or referral to hospital. On the other hand, a review of Verbakel et al. [8] stated that POC CRP test in ambulatory care accompanied by clinical guidance can reduce the immediate antibiotic prescribing rate, and another study showed that normal CRP levels discourage immediate antibiotic prescribing, even when EBM practice guidelines advise differently, and elevated CRP levels did not increase antibiotic prescribing [21]. Currently the EPI-centre team, led by Prof. Dr. Jan Verbakel, is performing a multicentre, cluster-randomized trial in ambulatory care, in which the impact of a diagnostic algorithm, including clinically guided POC CRP testing and safety netting advice on antibiotic prescribing rate and the further management of acutely ill children is investigated [50].

## Conclusion

In our study serious infections tended to have higher CRP values, however serious infections with CRP < 5 mg/L were also found. Exploring the POC CRP range in different diagnoses allowed us to examine its usefulness in stratifying acutely ill children in ambulatory care. A low POC CRP as a standalone tool did not seem to be sufficient to rule out serious infections, but its potential in assessing serious infections could increase when integrated in a clinical decision rule.

## Electronic supplementary material

Below is the link to the electronic supplementary material.

Supplementary Material 1

Supplementary Material 2

Supplementary Material 3

## Data Availability

The datasets generated and/or analysed during the current study are not publicly available but are available from the corresponding author on reasonable request.

## Abbreviations

CRP: C-reactive protein
DH: dehydration
ED: emergency department
GP: general practitioner
ICPC-2: second edition of the International Classification of Primary Care
INTEGO: integrated computerized morbidity registration network
IQR: interquartile range
metab: metabolic
nutrit: nutrition
NOS: not otherwise specified
POC: point-of-care
PVD: peripheral vascular disease
URTI: upper respiratory tract infections
UTI: urinary tract infection.
