# Supplementary material for: Point-of-care C-reactive protein test results in acute infections in children in primary care: an observational study
Source: BMC Pediatr. 2022 Nov 4;22:633. doi: 10.1186/s12887-022-03677-5 (PMC9635070; doi:10.1186/s12887-022-03677-5)
Supplement: Supplementary file 1 — Supplementary Material 1 [file 12887_2022_3677_MOESM1_ESM.docx]

**Appendix 1: Preliminary diagnoses: ranking, frequency and POC CRP (mg/L)**

| **ICPC-2 code** | **Number** | **Number Group known CRP** | **Median POC CRP** | **Min** | **Max** | **P25** | **P75** |
| --- | --- | --- | --- | --- | --- | --- | --- |
| **Acute URTI** | 2123 | 1536 | 9.0 | <5 | >200 | <5 | 23.00 |
| **Viral disease** | 1471 | 1284 | 9.0 | <5 | 170 | <5 | 24.00 |
| **Bronchitis/bronchiolitis** | 1083 | 918 | 11.0 | <5 | >200 | <5 | 26.00 |
| **Otitis media** | 993 | 741 | 15.0 | <5 | >200 | <5 | 36.00 |
| **Gastroenteritis** | 833 | 654 | 6.0 | <5 | >200 | <5 | 24.00 |
| **Pneumonia** | 451 | 411 | 33.0 | <5 | >200 | 12.00 | 67.00 |
| **Tonsillitis** | 401 | 281 | 39.0 | <5 | >200 | 14.00 | 69.00 |
| **Influenza** | 366 | 297 | 9.0 | <5 | 156 | <5 | 23.00 |
| **Dehydration** | 224 | 208 | 10.5 | <5 | >200 | <5 | 32.00 |
| **Asthma** | 196 | 143 | 6.0 | <5 | 81 | <5 | 18.00 |
| **Laryngitis/tracheitis** | 190 | 153 | <5 | <5 | 65 | <5 | 15.00 |
| **UTI** | 129 | 109 | 14.0 | <5 | 162 | <5 | 42.00 |
| **Pyelonephritis** | 106 | 104 | 48.0 | <5 | >200 | 21.75 | 120.25 |
| **Viral exanthem** | 88 | 66 | 8.0 | <5 | 80 | <5 | 25.25 |
| **Fever** | 87 | 69 | 32.0 | <5 | >200 | 8.00 | 62.00 |
| **Infectious disease** | 81 | 71 | 36.0 | <5 | 192 | 15.50 | 78.50 |
| **Conjunctivitis** | 77 | 51 | 12.0 | <5 | 81 | <5 | 39.50 |
| **Stomach function disorder** | 73 | 39 | <5 | <5 | 105 | <5 | 10.00 |
| **Serous otitis media** | 69 | 56 | 14.0 | <5 | 134 | <5 | 25.00 |
| **Sinusitis** | 63 | 48 | 14.0 | <5 | >200 | <5 | 40.00 |
| **Chickenpox** | 60 | 40 | 10.0 | <5 | 95 | <5 | 19.25 |
| **Mouth/tongue/lip disease** | 54 | 44 | 10.0 | <5 | 199 | <5 | 27.50 |
| **Strep throat** | 43 | 35 | 36.0 | <5 | 198 | 10.50 | 58.00 |
| **Nose symptom** | 36 | 27 | <5 | <5 | 99 | <5 | 9.00 |
| **Meningitis** | 35 | 30 | 9.0 | <5 | 76 | <5 | 29.00 |
| **Appendicitis** | 34 | 29 | 21.0 | <5 | >200 | <5 | 39.00 |
| **Lymphadenitis** | 31 | 28 | 28.0 | <5 | 123 | <5 | 59.25 |
| **Constipation** | 29 | 22 | <5 | <5 | >200 | <5 | <5 |

| **ICPC-2 code** | **Number** | **Number Group known CRP** | **Median POC CRP** | **Min** | **Max** | **P25** | **P75** |
| --- | --- | --- | --- | --- | --- | --- | --- |
| **Convulsion/seizure** | 28 | 25 | 8.0 | <5 | 114 | <5 | 24.00 |
| **Adverse effect medical agent** | 25 | 22 | 8.5 | <5 | 76 | <5 | 26.25 |
| **Skin infection** | 25 | 21 | <5 | <5 | 150 | <5 | 11.00 |
| **Allergic reaction** | 20 | 16 | <5 | <5 | 50 | <5 | <5 |
| **Ear discharge** | 19 | 14 | 8.0 | <5 | 95 | <5 | 25.75 |
| **Impetigo** | 15 | 11 | <5 | <5 | 95 | <5 | 6.50 |
| **Cough** | 14 | 8 | <5 | <5 | 21 | <5 | 7.00 |
| **Infectious mononucleosis** | 13 | 10 | 19.5 | <5 | 100 | 9.50 | 56.00 |
| **Bursitis/tendinitis/synovitis** | 11 | 10 | <5 | <5 | 49 | <5 | <5 |
| **Wheezing** | 11 | 9 | <5 | <5 | 25 | <5 | 12.00 |
| **Abdominal pain** | 11 | 9 | <5 | <5 | 22 | <5 | 5.00 |
| **Chronic obstructive pulmonary disease** | 11 | 8 | 13.5 | <5 | 86 | 9.50 | 24.00 |
| **Teeth/gum symptom** | 11 | 8 | 5.5 | <5 | 25 | <5 | 9.50 |
| **Complication of medical treatment** | 10 | 9 | 10.0 | <5 | 136 | 6.00 | 49.00 |
| **Oesophagus disease** | 10 | 6 | <5 | <5 | <5 | <5 | <5 |
| **Otitis externa** | 9 | 4 | 20.0 | <5 | 26 | 15.75 | 23.00 |
| **Urticaria** | 8 | 7 | <5 | <5 | 7 | <5 | 7.00 |
| **Vomiting** | 8 | 5 | 8.0 | <5 | 34 | <5 | 9.00 |
| **Disease digestive system, other** | 7 | 6 | 5.5 | <5 | 19 | <5 | 13.75 |
| **Lymphadenitis non-specific** | 6 | 6 | <5 | <5 | 60 | <5 | 20.50 |
| **Infections musculoskeletal system** | 6 | 6 | 15.0 | <5 | 71 | 7.50 | 42.00 |
| **Musculoskeletal disease, other** | 6 | 6 | <5 | <5 | 30 | <5 | 10.75 |
| **Lymph glands enlarged/painful** | 6 | 4 | <5 | <5 | 11 | <5 | 5.75 |
| **Hypertrophy tonsils/adenoids** | 6 | 3 | 49.0 | 10 | 50 | 29.50 | 49.50 |
| **Allergic rhinitis** | 6 | 3 | <5 | <5 | 41 | <5 | 22.50 |
| **Diarrhoea** | 6 | 3 | 5.0 | <5 | 11 | <5 | 8.00 |
| **Headache** | 6 | 5 | <5 | <5 | <5 | <5 | <5 |
| **Teeth/gum disease** | 5 | 5 | 11.0 | <5 | 26 | <5 | 21.00 |

| **ICPC-2 code** | **Number** | **Number Group known CRP** | **Median POC CRP** | **Min** | **Max** | **P25** | **P75** |
| --- | --- | --- | --- | --- | --- | --- | --- |
| **Neck symptom/complaint** | 5 | 4 | <5 | <5 | <5 | <5 | <5 |
| **Herpes simplex** | 5 | 4 | 7.0 | <5 | 33 | <5 | 15.75 |
| **Diaper rash** | 5 | 3 | <5 | <5 | 7 | <5 | 5.50 |
| **Migraine** | 5 | 2 | 6.0 | <5 | 8 | 5.00 | 7.00 |
| **Balanitis** | 4 | 4 | 5.0 | <5 | 25 | <5 | 10.75 |
| **Herpes zoster** | 4 | 3 | 6.0 | <5 | 8 | 5.00 | 7.00 |
| **Dermatitis contact/allergic** | 4 | 3 | 6.0 | 5 | 53 | 5.50 | 29.50 |
| **Purpura/coagulation defect** | 3 | 3 | <5 | <5 | 6 | <5 | 5.00 |
| **Cardiovascular disease other** | 3 | 3 | 76.0 | 9 | 158 | 42.50 | 117.00 |
| **Neurological symptom/complaint** | 3 | 3 | 65.0 | <5 | 74 | 34.50 | 69.50 |
| **Feeling anxious/nervous/tense** | 3 | 3 | <5 | <5 | 5 | <5 | <5 |
| **Boil/carbuncle** | 3 | 3 | <5 | <5 | 17 | <5 | 10.50 |
| **Eustachian salpingitis** | 3 | 2 | 20.0 | <5 | 36 | 12.00 | 28.00 |
| **Whooping cough** | 3 | 1 | 24.0 | 24 | 24 | 24.00 | 24.00 |
| **Epistaxis** | 2 | 2 | <5 | <5 | 5 | <5 | <5 |
| **Fainting/syncope** | 2 | 2 | <5 | <5 | <5 | <5 | <5 |
| **Poisoning by medical agent** | 2 | 2 | <5 | <5 | <5 | <5 | <5 |
| **Adverse effect physical factor** | 2 | 2 | 22.5 | <5 | 41 | 13.25 | 31.75 |
| **Anal fissure/perianal abscess** | 2 | 2 | 6.5 | 5 | 8 | 5.75 | 7.25 |
| **Infected finger/toe** | 2 | 2 | 22.0 | 20 | 24 | 21.00 | 23.00 |
| **Animal/human bite** | 2 | 2 | 64.5 | <5 | 125 | 34.25 | 94.75 |
| **Skin symptom/complaint other** | 2 | 2 | 34.5 | <5 | 65 | 19.25 | 49.75 |
| **Solar keratosis/sunburn** | 2 | 2 | <5 | <5 | <5 | <5 | <5 |
| **Endocrine/metab/nutrit. disorder** | 2 | 2 | 101.5 | 24 | 179 | 62.75 | 140.25 |
| **Glomerulonephritis/nephrosis** | 2 | 2 | 26.0 | <5 | 48 | 15.00 | 37.00 |
| **Abnormal urine test NOS** | 2 | 2 | 8.5 | <5 | 13 | 6.25 | 10.75 |
| **Orchitis/epididymitis** | 2 | 2 | <5 | <5 | <5 | <5 | <5 |
| **Sneezing/nasal congestion** | 2 | 1 | <5 | <5 | <5 | <5 | <5 |

| **ICPC-2 code** | **Number** | **Number Group known CRP** | **Median POC CRP** | **Min** | **Max** | **P25** | **P75** |
| --- | --- | --- | --- | --- | --- | --- | --- |
| **Weakness/tiredness general** | 2 | 1 | <5 | <5 | <5 | <5 | <5 |
| **Gastrointestinal infection** | 2 | 1 | <5 | <5 | <5 | <5 | <5 |
| **Mumps** | 2 | 1 | 33.0 | 33 | 33 | 33.00 | 33.00 |
| **Ear pain/earache** | 2 | 1 | <5 | <5 | <5 | <5 | <5 |
| **Rash localized** | 2 | 1 | <5 | <5 | <5 | <5 | <5 |
| **Shortness of breath/dyspnoea** | 1 | 1 | 53.0 | 53 | 53 | 53.00 | 53.00 |
| **Breathing problem, other** | 1 | 1 | <5 | <5 | <5 | <5 | <5 |
| **Foreign body nose/larynx/bronch** | 1 | 1 | 26.0 | 26 | 26 | 26.00 | 26.00 |
| **Congenital anomaly respiratory** | 1 | 1 | <5 | <5 | <5 | <5 | <5 |
| **Sweating problem** | 1 | 1 | <5 | <5 | <5 | <5 | <5 |
| **Blood/lymph/spleen disease other** | 1 | 1 | 29.0 | 29 | 29 | 29.00 | 29.00 |
| **Dyspepsia/indigestion** | 1 | 1 | <5 | <5 | <5 | <5 | <5 |
| **Change faeces/bowel movements** | 1 | 1 | <5 | <5 | <5 | <5 | <5 |
| **Worms/other parasites** | 1 | 1 | 6.0 | 6 | 6 | 6.00 | 6.00 |
| **Injury digestive system other** | 1 | 1 | <5 | <5 | <5 | <5 | <5 |
| **Chronic enteritis/ulcerative colitis** | 1 | 1 | 199.0 | 199 | 199 | 199.00 | 199.00 |
| **Eye appearance abnormal** | 1 | 1 | <5 | <5 | <5 | <5 | <5 |
| **Eye infection/inflammation other** | 1 | 1 | 25.0 | 25 | 25 | 25.00 | 25.00 |
| **Perforation ear drum** | 1 | 1 | 25.0 | 25 | 25 | 25.00 | 25.00 |
| **Atherosclerosis/PVD** | 1 | 1 | <5 | <5 | <5 | <5 | <5 |
| **Chest symptom/complaint** | 1 | 1 | <5 | <5 | <5 | <5 | <5 |
| **Arm symptom/complaint** | 1 | 1 | <5 | <5 | <5 | <5 | <5 |
| **Elbow symptom/complaint** | 1 | 1 | 12.0 | 12 | 12 | 12.00 | 12.00 |
| **Knee symptom/complaint** | 1 | 1 | <5 | <5 | <5 | <5 | <5 |
| **Ankle symptom/complaint** | 1 | 1 | <5 | <5 | <5 | <5 | <5 |
| **Foot/toe symptom/complaint** | 1 | 1 | <5 | <5 | <5 | <5 | <5 |
| **Fracture: other** | 1 | 1 | 13.0 | 13 | 13 | 13.00 | 13.00 |
| **Injury musculoskeletal NOS** | 1 | 1 | <5 | <5 | <5 | <5 | <5 |

| **ICPC-2 code** | **Number** | **Number Group known CRP** | **Median POC CRP** | **Min** | **Max** | **P25** | **P75** |
| --- | --- | --- | --- | --- | --- | --- | --- |
| **Malignant neoplasm nervous system** | 1 | 1 | 35.0 | 35 | 35 | 35.00 | 35.00 |
| **Concussion** | 1 | 1 | <5 | <5 | <5 | <5 | <5 |
| **Facial paralysis/bell's palsy** | 1 | 1 | <5 | <5 | <5 | <5 | <5 |
| **Acute stress reaction** | 1 | 1 | <5 | <5 | <5 | <5 | <5 |
| **Psychological symptom/complaint other** | 1 | 1 | <5 | <5 | <5 | <5 | <5 |
| **Lump/swelling localized** | 1 | 1 | <5 | <5 | <5 | <5 | <5 |
| **Rash generalized** | 1 | 1 | 8.0 | 8 | 8 | 8.00 | 8.00 |
| **Insect bite/sting** | 1 | 1 | <5 | <5 | <5 | <5 | <5 |
| **Pediculosis/skin infestation other** | 1 | 1 | 15.0 | 15 | 15 | 15.00 | 15.00 |
| **Dermatitis seborrhoeic** | 1 | 1 | 5.0 | 5 | 5 | 5.00 | 5.00 |
| **Ingrowing nail** | 1 | 1 | <5 | <5 | <5 | <5 | <5 |
| **Skin disease, other** | 1 | 1 | <5 | <5 | <5 | <5 | <5 |
| **Loss of appetite** | 1 | 1 | <5 | <5 | <5 | <5 | <5 |
| **Hypoglycaemia** | 1 | 1 | <5 | <5 | <5 | <5 | <5 |
| **Kidney symptom/complaint** | 1 | 1 | <5 | <5 | <5 | <5 | <5 |
| **Malignant neoplasm of kidney** | 1 | 1 | 60.0 | 60 | 60 | 60.00 | 60.00 |
| **Urinary calculus** | 1 | 1 | <5 | <5 | <5 | <5 | <5 |
| **Phimosis/redundant prepuce** | 1 | 1 | 5.0 | 5 | 5 | 5.00 | 5.00 |
| **Hydrocoele** | 1 | 1 | <5 | <5 | <5 | <5 | <5 |

*All ICPC-2 codes included in the study with their ranking based on frequency over alle cases, the median POC CRP (mg/L), the P25 and P75, and their frequency for the cases in which POC CRP was measured. ICPC-2, International Classification of Primary Care 2^nd^ edition; POC, point-of-care; CRP, C-reactive protein; URTI, upper respiratory tract infection; UTI, urinary tract infection; metab., metabolic; nutrit., nutrition; NOS, not otherwise specified; PVD, peripheral vascular disease.*
