# Supplementary material for: Point-of-care C-reactive protein test results in acute infections in children in primary care: an observational study
Source: BMC Pediatr. 2022 Nov 4;22:633. doi: 10.1186/s12887-022-03677-5 (PMC9635070; doi:10.1186/s12887-022-03677-5)
Supplement: Supplementary file 2 — Supplementary Material 2 [file 12887_2022_3677_MOESM2_ESM.docx]

| **Reasons for prescribing antibiotics** | **All settings** | **GP** | **Pediatric outpatient clinic** | **ED** |
| --- | --- | --- | --- | --- |
| Diagnosis | 743 (36.6%) | 283 (33.1%) | 267 (41.1%) | 193 (36.8%) |
| CRP value | 135 (6.7%) | 64 (7.5%) | 53 (8.2%) | 18 (3.4%) |
| Duration of fever | 131 (6.5%) | 93 (10.9%) | 31 (4.8%) | 7 (1.3%) |
| Seriously ill child | 106 (5.2%) | 91 (10.6%) | 11 (1.7%) | 4 (0.8%) |
| Findings on clinical examination | 89 (4.4%) | 70 (8.2%) | 8 (1.2%) | 11 (2.1%) |
| Medical history | 86 (4.2%) | 65 (7.6%) | 13 (2.0%) | 8 (1.5%) |
| High fever | 72 (3.5%) | 40 (4.7%) | 15 (2.3%) | 17 (3.2%) |
| Additional testing | 52 (2.6%) | 11 (1.3%) | 17 (2.6%) | 24 (4.6%) |
| Duration of illness | 49 (2.4%) | 39 (4.6%) | 3 (0.5%) | 7 (1.3%) |
| Age | 42 (2.1%) | 24 (2.8%) | 10 (1.5%) | 8 (1.5%) |
| Parents' expectation | 24 (1.2%) | 22 (2.6%) | 1 (0.2%) | 1 (0.2%) |
| Time of the consultation | 22 (1.1%) | 22 (2.6%) | 0 | 0 |
| Holiday plans | 18 (0.9%) | 17 (2.0%) | 0 | 1 (0.2%) |
| Gut feeling | 9 (0.4%) | 9 (1.1%) | 0 | 0 |
| Different ill child compared to previous episode | 5 (0.2%) | 5 (0.6%) | 0 | 0 |
| To prevent contamination | 3 (0.1%) | 3 (0.4%) | 0 | 0 |
| Social concerns | 1 (<0.1%) | 1 (0.1%) | 0 | 0 |
| **Total** | 2030 | 856 | 649 | 525 |
| Missing data | 850 | 257 | 315 | 278 |

**Appendix 2: Reasons for antibiotics prescribing per setting**

*Overview of the different reasons for prescribing antibiotics recorded by the physicians. GP, general practitioner; ED, emergency department; CRP, C-reactive protein.*
