# Supplementary material for: Point-of-care C-reactive protein test results in acute infections in children in primary care: an observational study
Source: BMC Pediatr. 2022 Nov 4;22:633. doi: 10.1186/s12887-022-03677-5 (PMC9635070; doi:10.1186/s12887-022-03677-5)
Supplement: Supplementary file 3 — Supplementary Material 3 [file 12887_2022_3677_MOESM3_ESM.docx]

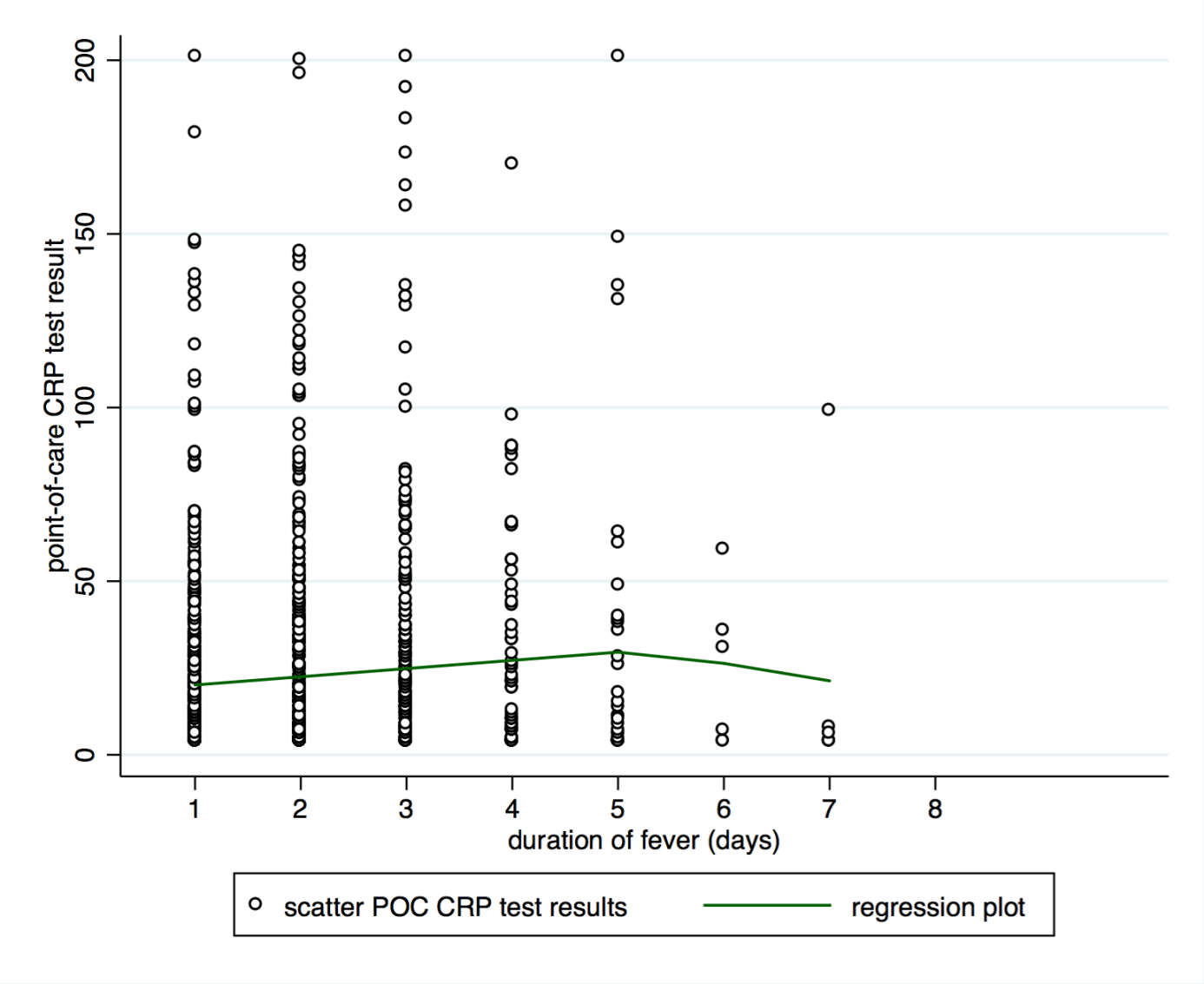


***Appendix 3:*** *Scatter plot of the POC CRP test results over time. When analyzed in relation with duration of fever, POC CRP did not differ significantly over time in our study****.****Verbakel JY, Lemiengre MB, De Burghgraeve T, et al. Validating a decision tree for serious infection: diagnostic accuracy in acutely ill children in ambulatory care. BMJ Open. 2015;5(8):e008657.*
